# The threads of history: why record your pandemic experiences for the RCPI archive?

**DOI:** 10.1017/ipm.2020.48

**Published:** 2020-05-22

**Authors:** Harriet Wheelock, Melissa Dickson, Elizabeth Barrett

**Affiliations:** Keeper of Collections, Royal College of Physicians of Ireland, Dublin 2, Ireland (Email: harrietwheelock@rcpi.ie); Department of English Literature, University of Birmingham, Birmingham, B15 2TT, UK; Children’s University Hospital Temple St, Dublin 1, Ireland; UCD Child and Adolescent Psychiatry, University College Dublin, Dublin 4, Ireland

Letter to the Editor,

We are living in a global crisis, in which the World Health Organization (WHO) officially recognised as a Pandemic on 11 March (WHO, 2020b). At the time of writing, in early April, with more than 2 million confirmed cases of coronavirus worldwide, a third of the global population on lockdown, and a rising death toll of over 137 000 (WHO, 2020a), many of us are confronting the vulnerabilities not only of our political, economic and public health systems, but also of our own bodies and minds, and those of our loved ones. At the same time that we face a tragic loss of life, often in extremely difficult circumstances, and without the touch or physical presence of loved ones, we are struggling with how that loss is to be grieved, remembered or even counted.

Some people will be disproportionately impacted by the necessity to quarantine. People with chronic illness may have to self-isolate for extended periods. In Ireland, anyone over 70 now faces this prospect. Brooks and colleagues reviewed 24 papers exploring the impact of this (Brooks *et al.* 2020), and they highlight that in the history of humanity, similar unknown pathogens have caused similar levels of difficulty. They note that the word ‘quarantine’ was first used in Venice in 1127 with regards to leprosy and referred to a widespread measure in tackling the impact of the ‘Black Death’. Later quarantines include the responses to severe acute respiratory syndrome and Ebola (Brooks *et al.* 2020).

Healthcare professionals are uniquely positioned to witness, experience and bear testimony to the realities experienced during quarantines, but those realities may also be lost in the effort to report on data. Often, in the course of a pandemic, what staff seek is practical support (around things like accommodation, social distancing and transport) and then support around supporting the mental health needs of patients, rather than for themselves (Lu *et al.* 2020). As Pfefferbaum and colleagues recognise, and as previous pandemics have demonstrated, there will also be a need for specialists with limited psychological medicine experience to be able to support their patients and colleagues (Pfefferbaum & North, 2020).

But what is the experience like on the ground? For example, in addition to supporting patients with COVID-19, other doctors including psychiatrists may be quite removed from their typical practice and ability to follow up with patients. They may be re-deployed to other settings. Personal Protective Equipment (PPE) availability is an issue, and PPE use may impact on communication. We live in an ‘always on’ world in a way that differs from previous epidemics – from Twitter and other social media to 24-hour news cycles. Is this helpful or unhelpful in sifting facts from fictions? How do we measure the impact of this? We know from previous disasters that distress is often ubiquitous and while many people are resilient, they experience adverse long-term health outcomes (Pfefferbaum & North, 2020). How is coping on the ground to be captured? There are of course many answers, but in 11th century Venice, it is a certainty that they were not captured by online questionnaires. Testimony and lived experience provided helpful historic records to complement and amplify the statistical data (Milne, 2018).

Similarly, how do we capture the grief of numerous families, easily obfuscated by daily statistics and factual briefings? Necessarily, global trends seek to capture the experience of the collective, anonymised and in the aggregate, rather than the individual. An alternative, qualitative medium is potentially helpful to future researchers – as they have been to researchers in the present – in the medical and humanities arenas to capture the spectrum of unique, often intensely personal experiences of this pandemic. These personal accounts may yield insights into the spread of this virus across the globe in which more formal documents would omit, and to consider those insights as they are inflected by such diverse issues as gender, race, sexuality and socio-economic status.

Narrative, and in particular, the diary and memoir provide an outlet for the personal perspective of the writer regarding those historical facts captured in official records. It allows the individual to express and explore their own experiences, while at the same time giving those experiences structure and lending nuance to larger historical forces. It is for these reasons that the Royal College of Physicians of Ireland (RCPI) Heritage Centre is currently working to build an archive of healthcare workers’ experiences of the pandemic.

As well as providing an outlet for individuals to express and perhaps in some ways, to work through their own experiences, the archive will create an invaluable resource for future researchers. The personal voice of the doctor, nurse or healthcare worker is often absent from the official record, creating a void in the historical narrative. Taking the 1918–19 flu pandemic as an example, there are only a handful of firsthand accounts by Irish medics in archives. The only contemporary account in the RCPI Archive is the diary of Kathleen Lynn (RCPI Archive, KL/1 – KL/2). This has presented problems for historians of the period, who have had to rely on the less personal official records, or accounts written decades afterwards. While these accounts can be extremely valuable, they do present some issues around how we remember events and the impact of hindsight on recollections. Dr Donough Macnamra touches on this in his account of the 1918–19 pandemic, ‘as it is now going on 40 years since that terrible time, my memory cannot hold much more than a general impression of the whole thing’ (Macnamara, 1954).

RCPI is interested in capturing a range of experiences, as they are communicated in the moment of the pandemic, in terms of their impact upon social, cultural and psychological structures and formations. This is an important addition to the historical archive. It is a rare thing in medicine to feel a connection to authors in Venice almost a thousand years ago. We urge our fellow clinicians to participate.

Interested clinicians are encouraged to contribute to this collection via heritagecentre@rcpi.ie.
